# Formulation and evaluation of curcumin microsponges for oral and topical drug delivery

**DOI:** 10.1007/s40204-018-0099-9

**Published:** 2018-09-21

**Authors:** Meenakshi Bhatia, Megha Saini

**Affiliations:** Drug Delivery Research Laboratory, Department of Pharmaceutical Sciences, Guru Jambheshwar University of Science and Technology, Hisar, 125001 India

**Keywords:** Curcumin, Microsponges, Quasi-emulsion solvent diffusion technique

## Abstract

The aim of the present study was to improve the release rate of curcumin by microsponges prepared through quasi-emulsion solvent diffusion technique using ethylcellulose and PVA as carriers. The microsponges were characterized by FTIR, DSC, XRD and SEM studies followed by determination of total drug content and entrapment efficiency. The prepared microsponges were further filled in hard gelatin capsule shell and then loaded in carbopol gel to evaluate its potential in oral and topical drug delivery. Further, it was observed from the studies on release rate that microsponges filled in hard gelatin capsule shells (batch MS4) showed 93.2% release of curcumin whereas pure curcumin filled in capsule showed only 11.7% release in 8 h study. Furthermore, the microsponges loaded in carbopol gel were evaluated for ex vivo drug deposition studies and it was found that 77.5% of the curcumin was released within 24 h. The estimated drug remained in the skin was 207.61 ± 5.03 μg/cm^2^ as determined by a Franz diffusion cell. The drug release profile data were found to be fitted best into the zero-order model with anomalous transport mechanism of drug release in both cases.

## Introduction

Microsponges are extremely cross-linked, non-collapsible, porous, polymeric microspheres having particle size range from 5 to 300 μm that can entrap wide range of active ingredients and release them over extended time (Osmani et al. 2015). Microsponges have unique dissolution and compression properties due to their sponge-like texture (Jangde 2011). They are highly effective, stable, non-irritant, non-toxic, non-allergic, non-mutagenic and also minimum side effects with improved patient compliance (Amrutiya et al. 2009). Various polymers like Eudragit RS100, ethylcellulose, polystyrene, PHEMA, etc. have been utilized in forming microsponges. Further, these active microsponges can be incorporated into formulations, such as capsules, gel and powders, and share a broad package of benefits (Pawar et al. 2015; Bothiraja et al. 2014; Li et al. 2013; Deshmukh and Poddar 2012; Jain and Singh 2011; Jain et al. 2011). The microsponges have demonstrated their use in cosmetics and pharmaceuticals viz. antifungal vaginal gel, in augmented arthritis therapy, as silver sulfadiazine-loaded microsponge gel for burn wounds, in gastroretentive delivery, as matrix tablet and in colon-specific drug delivery system, etc. (Salwa et al. 2018; Riyaz Ali et al. 2015; Kumar and Ghosh 2017; Arya and Pathak 2014; Mine et al. 2006).

Curcumin (CUR) is a yellow colored naturally occurring polyphenol compound obtained from the rhizomes of *Curcuma longa*, family *Zingiberaceae.* It is a highly pleiotropic molecule which can interact with different molecular targets involved in inflammation (Ornchuma et al. 2014). It can change inflammatory response by downregulating the activity of cyclooxegenase2 (COX2), lipoxygenase, and inducible iNOS (nitric oxide synthase) enzyme. Curcumin being a natural substance is non-toxic even at higher dose (Aziz et al. 2007).

The present study was designed with the objective to enhance the dissolution and thus the release rate of the drug and bioadhesive potential of the preparation. Curcumin-loaded microsponges were prepared by quasi-emulsion solvent diffusion method using ethyl cellulose (EC) and polyvinyl alcohol (PVA). The microsponges were characterized by FTIR, DSC, SEM and XRD studies. The prepared microsponges were filled in hard gelatin capsule shells and also loaded in carbopol gel. The capsules were evaluated by different pharmacopoeial tests and also the mechanical strength of the gel was determined by texture analyzer. The total drug content, production yield, mean particle size and entrapment efficiency were calculated. The formed microsponges were demonstrated for their applications in oral and topical delivery systems. The preparation was further evaluated for its in vitro drug release behavior and ex vivo bioadhesion studies using a Franz diffusion cell.

## Experimental

### Materials

Curcumin was obtained as a gift sample from Konark Herbal and Health Care, Mumbai, India. The reagents including ethyl cellulose were obtained from High Purity Laboratory Chemicals, Mumbai. Polyvinyl alcohol, dichloromethane, methanol, lactose monohydrate, magnesium stearate, triethanolamine, polyvinyl alcohol, propylene glycol, carbopol 934P and *N*-methyl-2-pyrrolidone were procured from SD Fine-Chem. Limited, Mumbai. Potassium dihydrogen orthophosphate anhydrous (monobasic), starch and acacia were obtained from Central Drug House (CDH, India). Disodium hydrogen phosphate dihydrate (Na_2_HPO_4_·2H_2_O) and sodium lauryl sulfate were provided by Hi-Media lab. Pvt. Ltd. All other chemicals were of reagent grades and used as procured.

### Methods

#### Preparation of curcumin microsponges

Curcumin (CUR) microsponges were prepared by quasi-emulsion solvent diffusion method (Jain and Singh 2010). The internal phase consisted of ethyl cellulose (2% w/v) in dichloromethane. The drug (100–500 mg) was gradually added to the EC solution with continuous stirring at 600 rpm. The internal phase was then added dropwise into the aqueous external phase containing polyvinyl alcohol (0.5% w/v) (Table 1). After 2 h of stirring, the microsponges were formed by evaporation of dichloromethane from the system. The microsponges were filtered and then dried in hot air oven at 40 °C till constant weight and stored in air tight container.

**Table 1:** Composition of different batches of microsponge formulations

Formulation	Curcumin (mg)	Ethyl cellulose (mg)	Dichloromethane (mL)	Polyvinyl alcohol (g)
MS1	100	100	5	0.5
MS2	200	100	5	0.5
MS3	300	100	5	0.5
MS4	400	100	5	0.5
MS5	500	100	5	0.5

### Characterization of microsponges

#### Particle size analysis

The mean particle size and polydispersity index (PdI) of all the batches of microsponges were measured using Mastersizer 2000 (Malvern Instruments Ltd.) at 25 °C.

#### Total drug content and entrapment efficiency

The weighed amounts of drug-loaded microsponges (10 mg) were dissolved in 10 mL methanolic phosphate buffer solution (pH 7.4) with occasional stirring. 1 mL of the above sample was appropriately diluted with methanolic phosphate buffer and the absorbance was taken at 428 nm against blank using methanolic phosphate buffer solution where the value of *E*_1%_ is 0.206. The total drug content was calculated as follows (Eq. 1):

1 $${\text{Total}}\;{\text{drug}}\;{\text{content}} = \frac{\text{Abs}}{{E_{1\% } }} \times {\text{dilution}}\;{\text{factor}} \times 10.$$

The drug entrapment efficiency (%) was calculated as (Eq. 2):

2 $$\% {\text{EE}} = {\text{TDC}}/{\text{amount}}\;{\text{of}}\;{\text{drug}}\;{\text{added}} \times 100,$$

where TDC is the total drug content in microsponges and %EE is the percentage of entrapment efficiency of the microsponges (Arya and Pathak 2014).

#### Fourier transform infrared spectroscopy (FTIR)

CUR, EC and CUR microsponges samples were subjected to Fourier transform infrared spectroscopy using KBr pellets in a Fourier transform infrared spectrophotometer (Perkin Elmer spectrum BX II) in the range from 4000 to 400 cm^−1^.

#### Differential scanning calorimetry (DSC) analysis

DSC analysis of CUR and CUR microsponges was carried out by heating the samples from 30 to 300 °C at a heating rate of 10 °C per min using DSC (SDT, Q600, TA instruments, USA).

#### Scanning electron microscopy (SEM)

The shape and surface of the CUR microsponges were examined using SEM (SEM, Environmental Scanning Electron Microscope model FEI Quanta 200F with Oxford-EDS system IE 250 × Max 80, The Netherlands) after coating. Prior to observation, the samples were mounted on metal grids, using double-sided adhesive tape and coated with gold under vacuum.

#### X-ray diffraction (XRD) study

The CUR, EC and CUR microsponges powder samples were scanned using an X-ray diffractometer (Miniflex 2, Rigaku, Japan) from 0° to 50° diffraction angle (2*θ*) range under the following measurement conditions: source, nickel filtered CuKα radiation; voltage 35 kV; current 25 mA; scan speed 0.05 min^−1^, division slit 1.25°, receiving slit 0.3 mm.

### Stability study

The stability studies of CUR microsponges were carried out in accelerated conditions as per ICH guidelines. The microsponge formulations were kept at 40 °C ± 2 °C and 75% ± 5% RH for 3 months. After 3 months, microsponges were analyzed for physical appearance, in vitro drug release and FTIR spectroscopy.

### Evaluation of capsules containing CUR microsponges

After filling the CUR microsponges in capsule shells along with the excipients starch, acacia, sodium lauryl sulfate, lactose monohydrate and magnesium stearate, the filled capsules were evaluated for their organoleptic properties (size, shape and color, etc.). The weight variation and the disintegration tests were performed on filled capsules as per the methods specified in IP 2007. All the experiments were performed in triplicate.

#### In vitro drug release study

In vitro release rate study of CUR microsponges filled in capsule shell was carried out using a USP type I dissolution apparatus (TDL-08L Electrolab, India). Each jar was provided with one capsule in 900 mL of dissolution media, i.e., methanolic phosphate buffer (pH 7.4) and maintained at temperature of 37.0 ± 0.5 °C with continuous stirring at 50 rpm for 8 h. An aliquot of 5 mL sample was withdrawn at different time intervals and the same volume was replaced with fresh methanolic phosphate buffer. The content of CUR released was spectrophotometrically analyzed by measuring the absorbance at *λ*_max_ of 428 nm. The mechanism of drug release from the capsules was determined by fitting the release data into several release kinetic models like zero-order, first-order, and Higuchi and Korsmeyer/Peppas plots (Higuchi 1961, Mathew et al. 2009).

### Preparation of gel containing curcumin microsponges

Carbopol 934P (1% w/v) was initially soaked in water for 2 h and homogenously dispersed by agitation at 600 rpm using magnetic stirrer. Curcumin microsponges were then uniformly dispersed in carbopol gel. Triethanolamine (2% v/v) was added to neutralize the pH. To this aqueous dispersion, propylene glycol and *N*-methyl-2-pyrrolidone were added as permeation enhancers.

### Evaluation of gel containing CUR microsponges

After visual examination of the gel for its consistency, color and homogeneity, the gel was further evaluated for following parameters.

#### pH determination

The pH of the prepared gel was measured using pH meter (standardized using buffer, pH 7 before use) by putting the tip of the electrode into the gel and after 2 min the result was recorded. The measurement of pH of formulation was done in triplicate and the mean value was calculated.

#### Spreadability

Spreadibility of the gel was determined after placing a weighed amount of sample between 2 glass slides and a weight of 500 g was kept over the slides for about 5 min after which no more spreading was expected. Diameters of spread circles (initial and final) were measured in cm and were taken as comparative values for spreadability.

#### Viscosity

Rheology includes the measurement of viscosity in centipoise, which indicates resistance of a fluid to flow. The viscosity of gel was measured by Brookfield viscometer using spindle No. 7 at different rpm at room temperature.

#### Mechanical characterization of gels

Structural analysis of carbopol gel and curcumin microsponges gel was done to determine their mechanical properties such as hardness, cohesiveness, and adhesiveness. Mechanical characterization was conducted employing a software-controlled penetrometer (TA-XT2, Stable Micro Systems, UK) by measuring the resistance to penetration and withdrawal of the needle probe (diameter 2 mm) with pre-test speed of 1.5 mm/s, test speed 1.0 mm/s and post-test speed of 10 mm/s. The needle probe (2 mm) was compressed down into the samples at a constant rate of 1.0 mm/s to a depth of 8 mm. The study was carried out in triplicate and the results were expressed as mean ± SD. From the resulting texturogram, hardness and adhesiveness were calculated (Bhatia and Ahuja 2013).

#### Ex vivo drug deposition studies

Drug deposition study was performed on the excised rat abdominal skin using a Franz diffusion cell (Padamwar and Pokharkar 2006). Epidermal side of the skin was exposed to ambient condition, while dermal side was kept facing the receptor solution. Receptor compartment containing 30 mL phosphate buffer pH 7.4 was thermostated at 37 ± 0.5 °C and stirred at 600 rpm. The skin was saturated with diffusion medium for 1 h before the application of sample. A 100 mg of gel sample (equivalent to 30 mg curcumin) was applied on the donor compartment. The samples were withdrawn at different time intervals. For determination of drug deposited in the skin, the diffusion cell was dismantled at the end of the run (24 h). The skin was carefully removed and drug present on the skin surface was cleaned with distilled water and analyzed for drug content.

#### Quantification of curcumin from the skin samples

Drug was extracted from the skin using a modified procedure (Echevarria et al. 2003). Briefly, the skin was cut into small pieces and homogenized with 10 mL phosphate buffer pH 7.4 by tissue homogenizer. The homogenized sample was subjected to ultrasonication for 10 min for complete extraction of drug. This solution extract was centrifuged at 5000 rpm for 10 min. The supernatant was collected and analyzed by UV spectroscopy by measuring absorbance at wavelength of 428 nm.

#### Data and statistical analysis

The steady-state flux (*J*, μg/cm^2^/h) was calculated from the slope of linear plot of the cumulative amount permeated per unit area (μg/cm^2^) as a function of time (h). The lag time (*t*_L_, h) was determined from the *x*-intercept of the slope at steady state. The permeability coefficient (KP, cm^2^/s) was calculated from the flux and donor drug concentration (Amrutiya et al. 2009).

## Result and discussion

Quasi-emulsion solvent diffusion method has been used for the development of CUR microsponges. These microsponges were filled in capsule shells and were also loaded in carbopol gel. The microsponges were characterized by various parameters and evaluation of the capsules and gel was carried out by different techniques.

### Characterization of microsponges

#### Particle size analysis

The production yield (%) and mean particle size of microsponges are shown in Table 2. It is found that on increasing the drug to polymer ratio, the increase in production yield and mean particle size is observed up to batch MS4, that is having drug to polymer ratio (4:1), but as the drug to polymer ratio is increased (5:1) the decrease in production yield and entrapment efficiency is observed. This may probably be due to lesser availability of polymer to entrap the drug and the growth in mean particle size may be due to increasing drug to polymer ratio.

**Table 2:** Effect of drug to polymer ratio on various parameters

Formulation code	Drug/polymer ratio	Total drug content (%)	Production yield (% ± SD)	Mean particle size (µm)	Entrapment efficiency (%)
MS1	1:1	24.5 ± 2.9	57.3 ± 1.5	97.4 ± 2.9	21.3 ± 2.7
MS2	2:1	32.2 ± 3.5	66.6 ± 2.8	103.5 ± 1.8	30.2 ± 2.3
MS3	3:1	59.8 ± 3.3	85.3 ± 2.3	163.4 ± 2.7	55.8 ± 2.5
MS4	4:1	97.2 ± 1.5	95.2 ± 1.3	183.6 ± 2.3	93.2 ± 2.4
MS5	5:1	64.7 ± 2.1	77.2 ± 3.2	196.9 ± 3.0	61.7 ± 2.2

#### Total drug content (TDC) and entrapment efficiency (EE)

TDC and EE in different formulations were estimated by UV spectrophotometric method. The total drug content and entrapment of the drug depend on the successful molecular association of the drug with the polymers. TDC and EE of the microsponges were found in the range of 24.5 ± 2.9 to 97.2 ± 1.5% and 21.3 ± 2.7 to 93.2 ± 2.4% of different batches (Table 2). The values of TDC and EE were found maximum for the formulation MS4 having the drug to polymer ratio of 4:1. A drop in TDC and EE was observed on further increasing drug/polymer ratio. The probable reason for this decrease in TDC and EE could be that the optimum concentration of polymer is not available to coat or entrap the drug molecules.

#### Fourier transform infrared spectroscopy

**Fig. 1:**
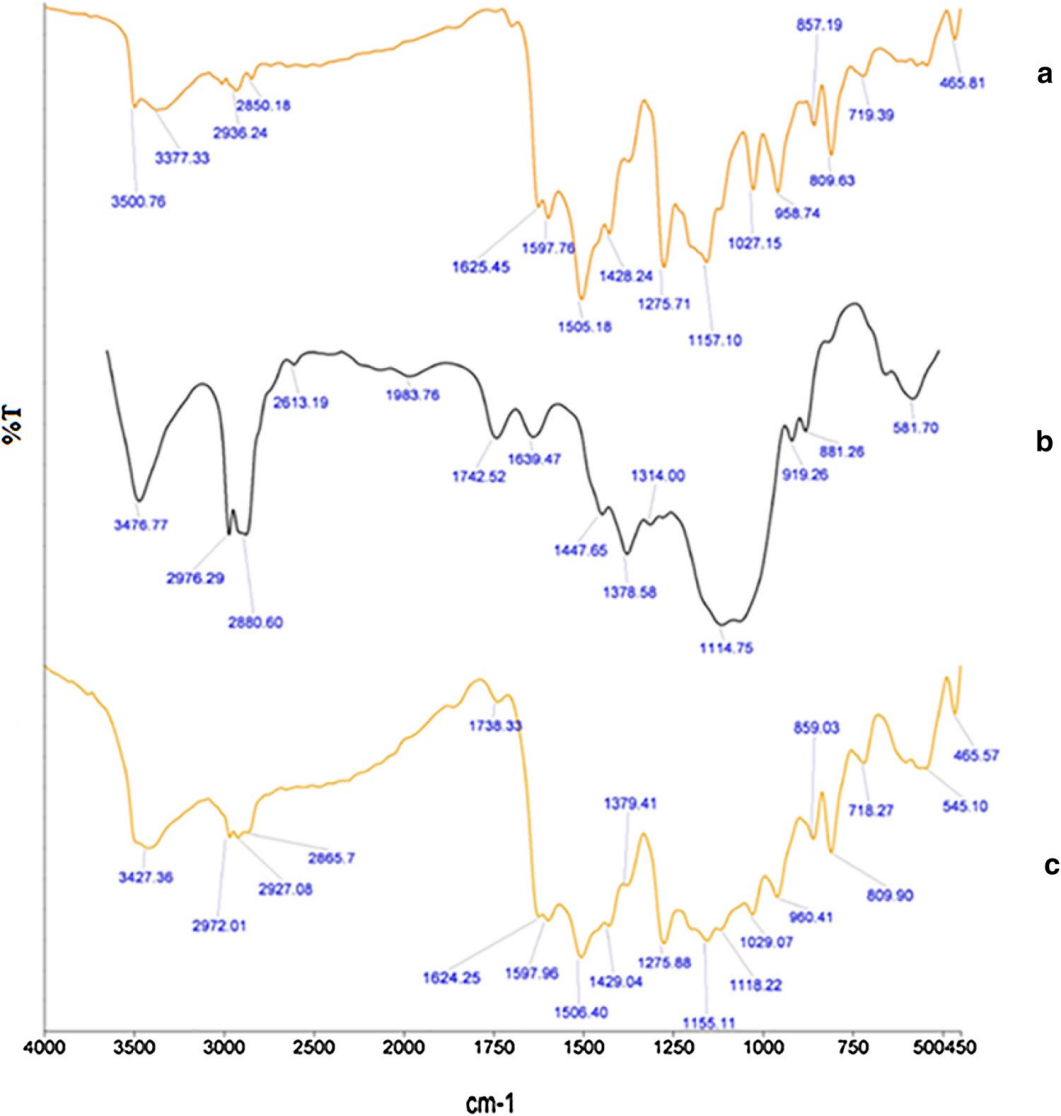
FTIR spectra of (a) CUR, (b) EC and (c) CUR microsponges

Figure 1 exhibits the FTIR spectra of CUR, EC and CUR microsponge. In spectra of CUR, as shown in Fig. 1a, the characteristic transmittance bands were observed at 1597, 1505, 1275, and 1157 cm^−1^ corresponding to stretching –C=C vibrations of benzene, aromatic –C–O stretching of (–OMe and –OH), and –C–O–C stretching (–OMe). Further, characteristic bands for phenolic –OH and conjugated ketonic –C=O vibrations were observed at 3500 cm^−1^. The spectra of EC, Fig. 1b, show the characteristic absorption of alcoholic hydroxyl groups at 3476.71 cm^−1^; the continuous several absorption peaks in range of 2900–2800 cm^−1^ and 1300–1000 cm^−1^ represent the ethoxyl groups while the spectra of CUR microsponges (Fig. 1c) show broadening of band at around 3400 cm^−1^ and characteristic bands in the range 1597–950 cm^−1^. However, comparison of the spectra demonstrated no new characteristic peaks in the microsponge which indicated no physical or chemical interactions between curcumin and carrier polymer.

#### Differential scanning calorimetry

**Fig. 2:**
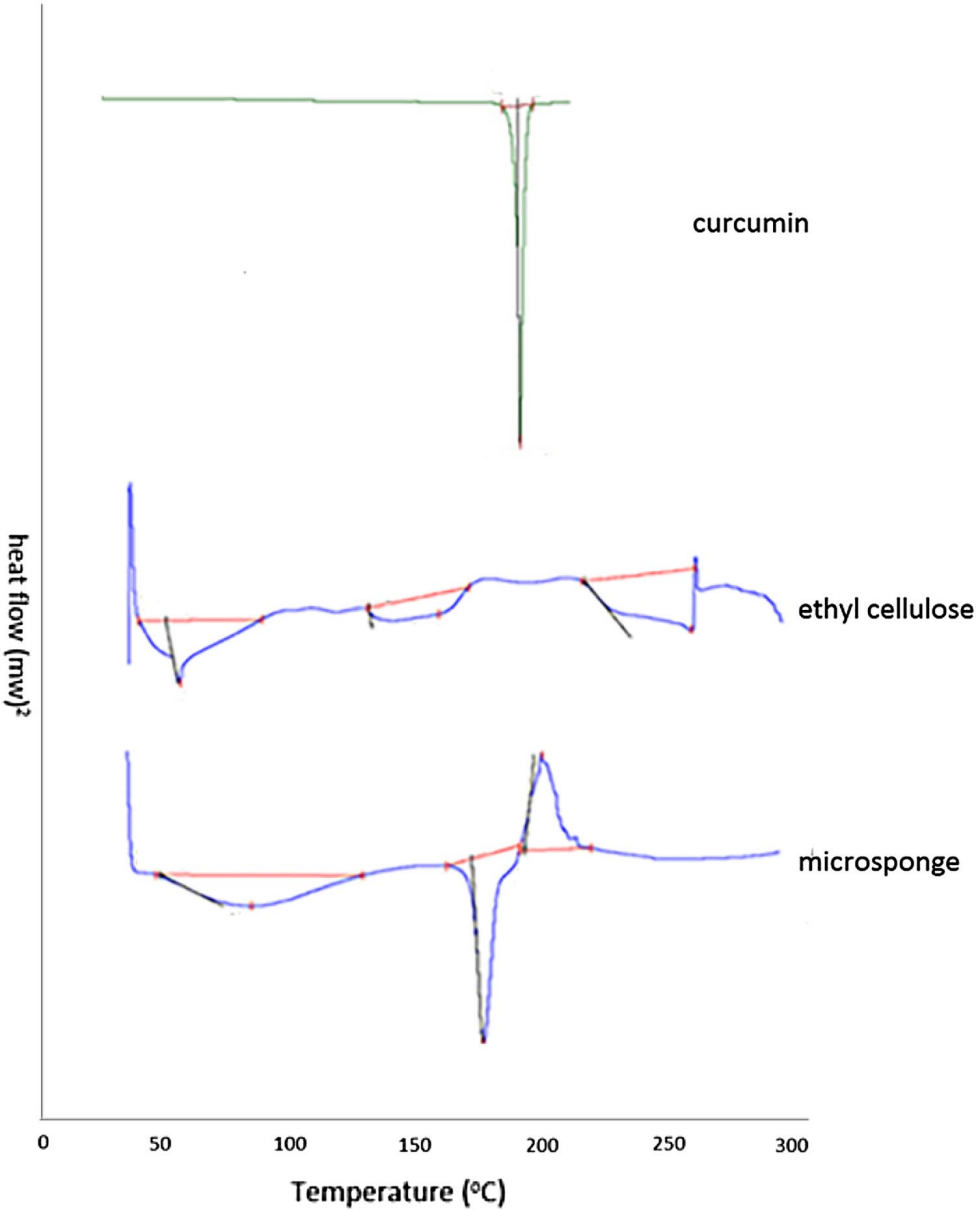
DSC of curcumin, ethyl cellulose and microsponge

Figure 2 represents the thermogram of CUR, EC and CUR microsponges. Ethyl cellulose being an amorphous substance does not possess sharp endothermic peaks whereas the thermogram of CUR shows a sharp endotherm at 184.48 °C with heat of fusion of 137.1 J/g corresponding to its melting point. The thermogram of curcumin microsponges shows a broad endotherm at 82.83 °C, a sharp endotherm at 176.57 °C and an exotherm at 199.933 °C with heat of fusion of 83.56 J/g, 82.81 J/g and 57.93 J/g, respectively. The shifting of endotherms and appearance of a new exotherm and decrease in heat of fusion indicate that some modifications have occurred.

#### Scanning electron microscopy

**Fig. 3:**
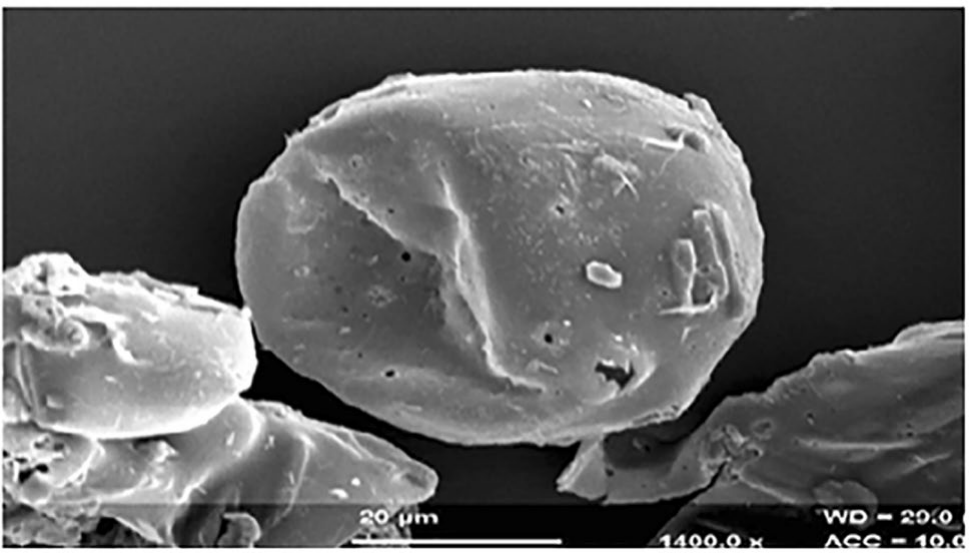
SEM image of CUR microsponges

Figure 3 exhibits the SEM showing surface morphology of CUR microsponges. SEM micrographs displayed that microsponges formed are predominantly spherical and entire curcumin crystals are not seen.

#### X-ray diffraction study

**Fig. 4:**
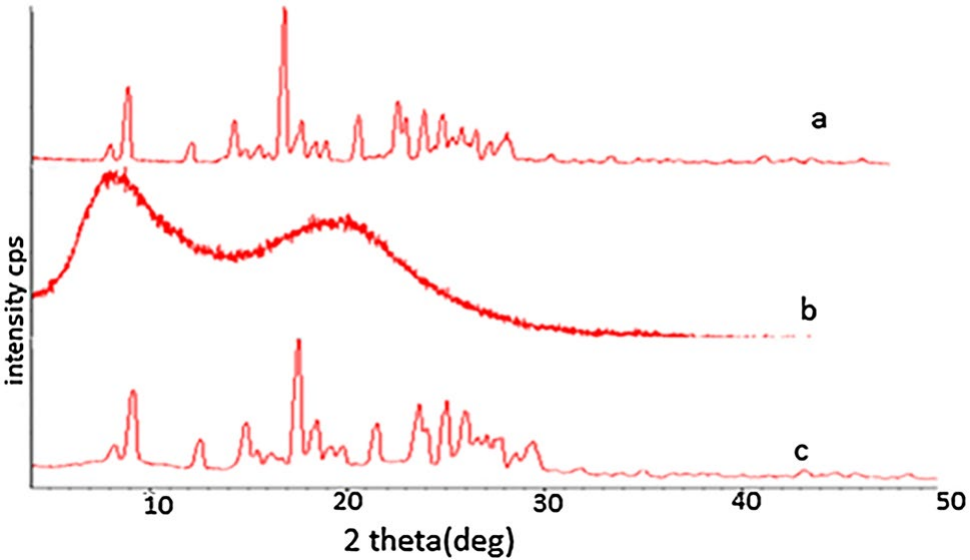
XRD spectra of (a) CUR, (b) EC and (c) CUR microsponges

Figure 4 displays the X-ray diffraction spectra of CUR, EC and CUR microsponge. X-ray diffractogram of EC is typical of amorphous materials with no sharp peaks. The spectra of CUR show sharp peaks at 17°, 22°, 24°, 30° (2*θ*) indicating the crystalline nature of curcumin, while the diffractogram of CUR microsponge shows sharp peaks at 18°, 24°, 38° (2*θ*) with decreased intensity which indicates decrease in crystalline nature of the microsponge.

### Stability study

The batch MS4 was subjected to 3-month stability study at accelerated conditions and was analyzed for physical appearance, in vitro drug release and FTIR spectroscopy. After 3 months, the formulation was found with no significant change in its appearance. The in vitro percentage drug release came out to be 91.45 similar and also the FTIR spectra revealed no sign of instability. Thus, all these parameters suggested that the formulation MS4 may have good shelf life.

### Evaluation of capsules

The average weight of capsule formulations was found to be within pharmacopoeial limit. The disintegration of the capsule formulations took to be in the range of 17–25 min that is also within the specified limits as the maximum disintegration time for capsule is 30 min.

#### In vitro drug release study

**Fig. 5:**
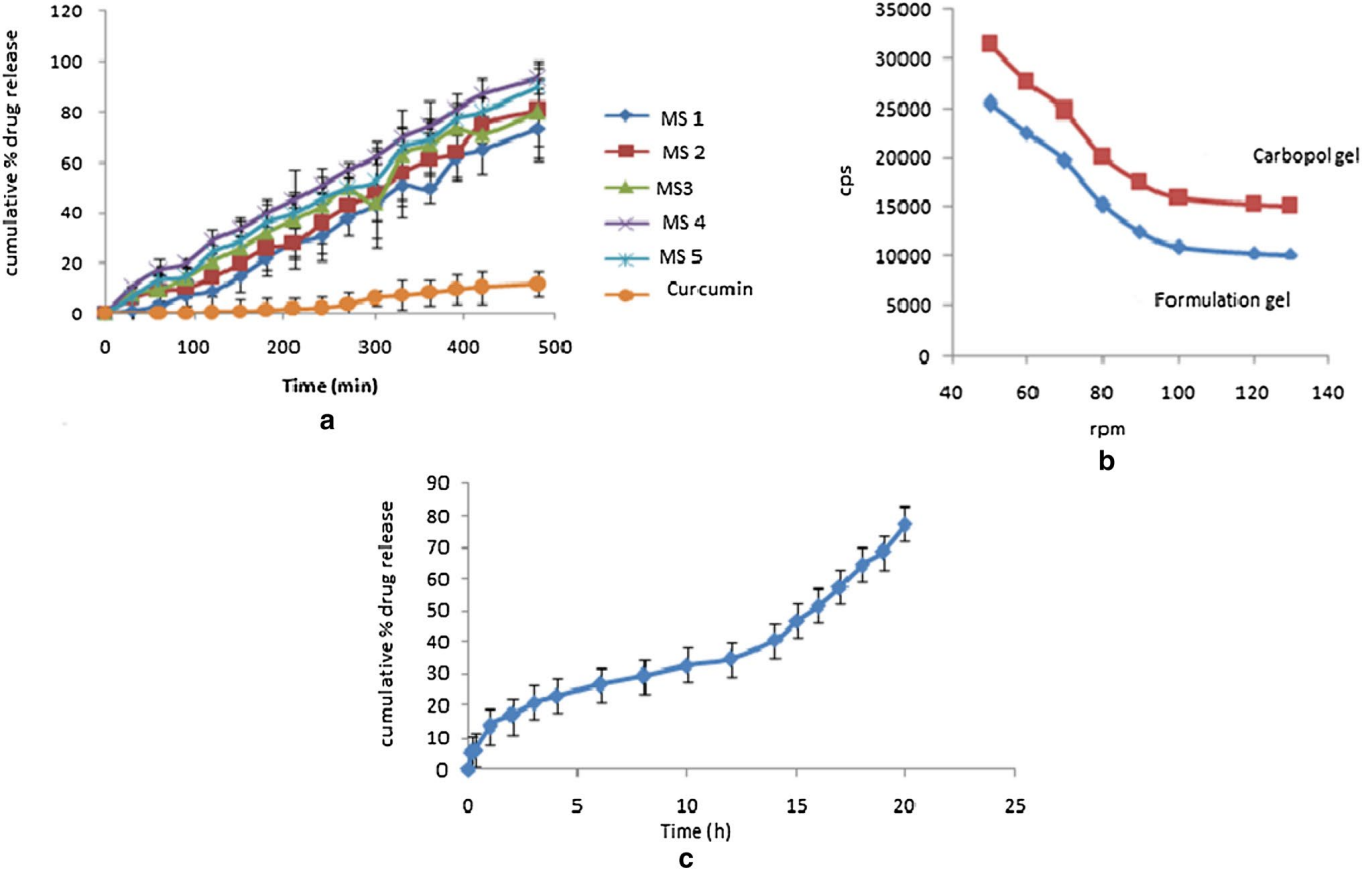
**a** In vitro drug release profile of curcumin, **b** viscosity of carbopol gel and curcumin microsponge gel, **c** ex vivo drug release profile of curcumin

All the batches of CUR microsponges-loaded capsules were evaluated for drug release behavior. Figure 5a displays the in vitro release profile of CUR from various batches using USP type I dissolution apparatus. The drug release was determined in methanolic phosphate buffer (pH 7.4) maintained at temperature of 37.0 ± 0.5 °C and stirred at 50 rpm for 8 h. It can be inferred from the graph that the batch MS4 shows 93.2% release for curcumin in microsponges, whereas the equivalent amount of pure CUR shows only 11.7% of release in 8 h. Therefore, it is inferred from the study that microsponges2 have displayed enhanced dissolution of curcumin as compared to pure drug. The release rate data of CUR microsponges-loaded capsules were fitted into various kinetics models to estimate their release kinetics and mechanism of release. The release kinetics data were found to be fitted best into the zero-order model (*R*^2^ = 0.996) for curcumin in microsponges with anomalous transport mechanism of drug release.

### Evaluation of microsponge gel

#### Visual examination

The non-opaque pale yellow gel with a pH of 6.42 ± 0.415 was formed with no-phase separation that indicated the homogeneity of the preparation.

#### Spreadability and viscosity

An apparatus suggested by Mutimer et al. (1956) modified suitably in the laboratory and was used for spreadability study. The result of spreadability is shown in Table 3. Viscosity of carbopol and microsponge gel was determined using Brookfield viscometer. There is no significant difference in viscosity of both the gels and a drop in viscosity is observed with the increasing rpm for both the gels as shown in Fig. 5b.

**Table 3:** Evaluation of spreadability, hardness and adhesiveness of gels

Formulation	Spreadability (diameter in cm)	Hardness (g)	Adhesiveness (g.s.)
Carbopol gel	2.6	3.85 ± 0.36	17.43
Formulation gel	1.8	3.40 ± 0.1	16.08

#### Mechanical characterization

**Fig. 6:**
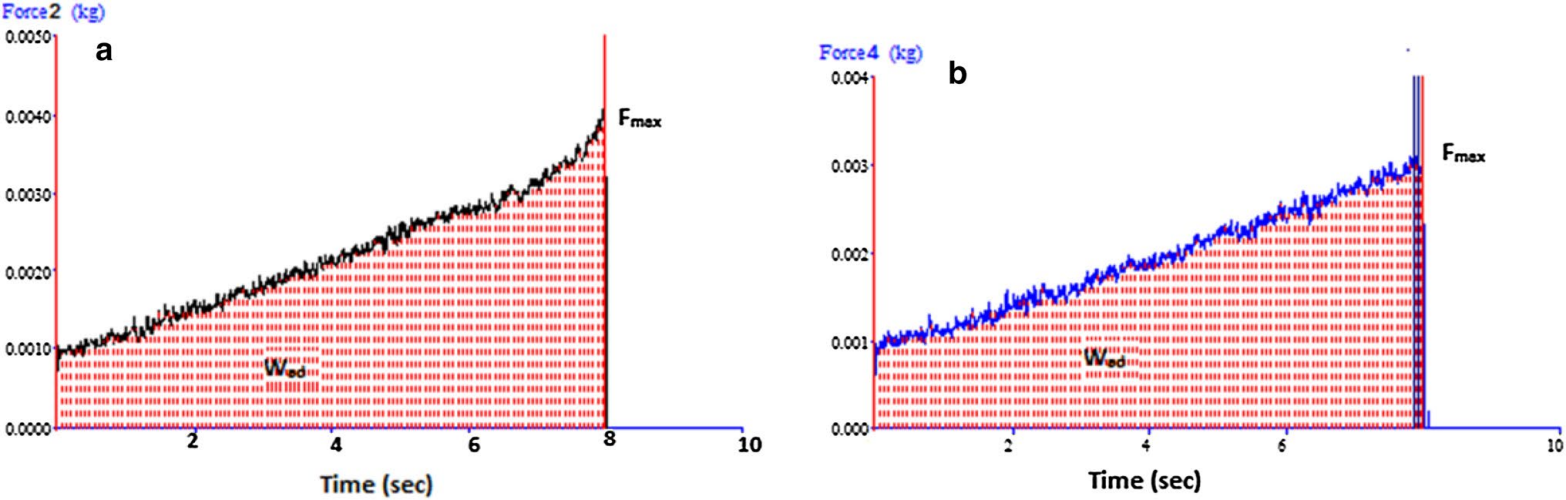
Texture analysis of **a** carbopol gel and **b** CUR microsponges gel

The topical formulation must exhibit acceptable mechanical characteristics such as low hardness and high adhesiveness. The maximum detachment force; *F*_max,_ i.e., hardness and work of adhesion; W_ad_ is determined by the plots of force versus distance data for carbopol gel and microsponges gel using texture analyzer. The height of the peak is the maximum force required to separate the probe from the gel (i.e., *F*_max_) and the total amount of forces involved in the probe withdrawal from the gel (*W*_ad_) is calculated from the area under the force versus distance curve. The texturograms as shown in Fig. 6a, b displayed hardness and work of adhesion of carbopol gel and microsponges gel, respectively. It was observed from Fig. 6a that hardness, i.e., *F*_max_ is 3.85 ± 0.36 g and work of adhesion is found to be 17.43 g.s. for carbopol gel, whereas the Fig. 6b shows the hardness and work of adhesion are 3.4 ± 0.1 g and 16.08 g.s. of microsponges gel. Hence, no significant difference is observed in the hardness and adhesiveness of the gels.

#### Ex vivo drug deposition studies

Figure 5c shows the ex vivo drug release profile of curcumin indicating that microsponge-loaded gel improved the drug residence time in skin and therapeutic drug concentrations were maintained for a prolonged period with 77.5% drug release in 24 h. At the end of the study, the drug (CUR) remained in the skin was found to be 207.61 ± 5.03 μg/cm^2^ as determined by homogenizing the skin with 10 mL phosphate buffer pH 7.4. This solution extract was centrifuged and the supernatant was analyzed at a wavelength of 428 nm using UV–Vis spectroscopy. The permeation flux and permeability coefficient are found to be 10.876 μg cm^−2^ h^−1^ and 0.3625 × 10^−6^ cm^2^ h^−1^, respectively, calculated from the slope of a linear plot of the cumulative amount permeated per unit area (μg/cm^2^) as a function of time (h). This ex vivo drug release profile data were found to be fitted best into the zero-order model (*R*^2^ = 0.921) with anomalous transport mechanism of drug release.

## Conclusion

The objective of developing polymeric microsponge delivery system was to deliver curcumin in a sustained manner for an extended period of time, to reduce frequency of administration and to improve its bioavailability. Therefore, in the present study curcumin microsponges were prepared by simple, reproducible and rapid quasi-emulsion solvent diffusion method. The formulation was characterized by FTIR, DSC, SEM, and XRD studies. The prepared microsponges were then incorporated in capsule dosage form and were loaded in carbopol gel. Varied drug–polymer ratio reflected a remarkable effect on particle size, total drug content and encapsulation efficiency. The batch MS4 possesses the maximum TDC of 97.2% and entrapment efficiency of 93.2% with a production yield of 95.2%. The curcumin microsponges filled in the capsule shells showed 93.2% release of curcumin in 8 h study following zero-order release kinetics with anomalous transport mechanism of drug release. Further, the curcumin microsponges loaded in the carbopol gel showed 77.5% of drug release in 24 h as determined by the ex vivo drug release profile study carried out using a Franz diffusion cell. The amount of drug remained in the skin was found to be 207.61 ± 5.03 μg/cm^2^. The drug release profile data were found to be fitted best into the zero-order model (*R*^2^ = 0.921) with anomalous transport mechanism of drug release. Thus, curcumin microsponges prepared in this study were found to be promising as newfangled delivery system offering prolonged release of drug and, hence, would be more useful than conventional formulation therapy in oral as well as in topical drug delivery.
